# Validation of the *Brugia* Test Plus to detect IgG4 antibodies in individuals from Belitung Timur, a *Brugia malayi* endemic area in Indonesia

**DOI:** 10.1371/journal.pntd.0013449

**Published:** 2025-08-20

**Authors:** Taniawati Supali, Elisa Iskandar, Noviani Sugianto, Yenny Djuardi, Katherine Gass, Jean M. Saunders, Peter U. Fischer, Marco A. Biamonte

**Affiliations:** 1 Department of Parasitology, Faculty of Medicine, Universitas Indonesia, Jakarta, Indonesia; 2 Coalition for Operational Research on Neglected Tropical Diseases, Decatur, GeorgiaUnited States of America; 3 Drugs and Diagnostics for Tropical Diseases, San Diego, California, United States of America; 4 Infectious Diseases Division, Department of Medicine, Washington University School of Medicine, Saint Louis, Missouri, United States of America; University of Agricultural Sciences and Veterinary Medicine Cluj-Napoca, Life Science Institute, ROMANIA

## Abstract

The *Brugia* Test Plus (BT+) is a new rapid diagnostic test for *Brugia* species which detects human IgG4 antibodies specific for the immunogenic *Brugia* protein BmR1. The aim of this study was to evaluate the BT+ assay with several types of sample-matrices: whole blood, plasma, and dried blood spots (DBS) from individuals living in Belitung Timur, a *Brugia malayi* endemic area in Indonesia. Night blood was collected from residents living in four presumed endemic villages, while DBS were collected from schoolchildren living in those four villages. The sensitivity of BT+ was measured by comparing the BT+ results to microscopic examination for microfilaria (Mf). The sensitivity of BT+ with whole blood under field conditions was 84.9% (95% CI 68.1–94.9, n = 33), and with EDTA plasma under laboratory conditions was 95.9% (95% CI 88.5–99.1, n = 73). Specificity was not assessed as it is impossible do so for an antibody test in endemic areas. In Mf-negative individuals, BT+ detected IgG4 antibodies in 204 out of 1,547 adult plasma samples (13.2%) and 7 out of 146 plasma samples collected from children aged 10–16 (4.8%). Detection of IgG4 antibodies in DBS collected from first and second grade schoolchildren (age 6–8) showed that only 1 out of 244 schoolchildren was positive (0.1%). Three individual readers were responsible for reading the BT+ . Statistical analysis showed high agreement among those readers (Kappa agreement value above 0.9). Laboratory technicians found the BT+ is simple to perform, easy to interpret the results, and appreciated the small volume of blood required (5 µL). This study has demonstrated that the novel BT+ is feasible for teams to implement and achieves good sensitivity, making it well suited for monitoring and evaluating the progress of the brugian- filariasis elimination program.

## Author summary

The *Brugia* Test Plus (BT+) is a new rapid diagnostic test for *Brugia* species that detects human IgG4 antibodies directed against the *Brugia* protein BmR1. BT+ was evaluated in the field using a variety of samples, including whole blood, plasma, and dried blood spots (DBS) from people living in Belitung Timur, an endemic area for *Brugia malayi* in Indonesia. Night blood samples were collected from residents of four endemic villages, and day blood samples for DBS were obtained from schoolchildren in same villages. The sensitivity of BT+ was determined by comparing its results to a microscopic test for microfilaria (Mf). BT+  had a sensitivity of 84.9% with whole blood in the field and 95.9% with EDTA plasma in the lab. Specificity was not evaluated in *B. malayi* endemic area. Overall, the BT+ was found to be straightforward to perform and interpret, and its analytic performance is well suited for measuring the impact of the *brugian*-filariasis elimination program.

## Introduction

Lymphatic filariasis (LF) caused by the three filarial species, *Wuchereria bancrofti*, *Brugia malayi*, and *Brugia timori,* is a public health problem in Indonesia. According to reports, LF is endemic in 236 out of 514 districts or cities in Indonesia, with *Brugia* *malayi* being the dominant parasite, which is endemic in 189 of these areas [1]. Infections caused by these filarial worms result in lifelong disabilities, leading to physical and psychological distress as well as significant socio-economic disruption for the affected individuals [2].

To combat LF, the World Health Organization (WHO) launched a global elimination program with the goal of eliminating the disease by 2030 [3]. A Mass Drug Administration (MDA) strategy was implemented to achieve the goal, with the current WHO recommendation being a triple drug therapy of ivermectin (200 µg/kg body weight), diethylcarbamazine citrate (DEC, 6 mg/kg body weight), and albendazole (400 mg). This combination is administered annually for two consecutive years and was introduced as a replacement for the previous regimen of DEC 6 mg/kg body weight and albendazole 400 mg, which required five years of administration [4].

Indonesia has been actively participating in the global LF elimination program since 2002, implementing MDA with DEC 6 mg/kg body weight and albendazole 400 mg annually for at least five consecutive years among residents of endemic districts or cities. To evaluate the success of the elimination program, it is crucial to use diagnostic tools that are sensitive, specific, reliable, and suitable for field use. The detection of anti-filarial IgG4 antibodies, which serve as markers of active *Brugia* infection or exposure, has been recommended by WHO for monitoring and evaluating LF elimination efforts in *Brugia* endemic areas [5–7].

However, the current serological diagnostic tool (*Brugia* Rapid Test) [8] widely used for monitoring and evaluating *brugian* filariasis elimination programs has shown inconsistent results in detecting infected individuals. This inconsistency is mainly due to significant lot-to-lot variability in sensitivity. Therefore, it is essential to develop diagnostic tools with improved consistency for reliable detection of IgG4 antibodies in communities following mass treatment.

A new diagnostic tool, *Brugia* Test Plus (BT+), developed by Drugs & Diagnostics for Tropical Diseases, San Diego, California, has been previously tested at Washington University School of Medicine in St. Louis using 65 archived plasma samples from individuals with *B. timori* microfilaremia from Alor (Indonesia) and 36 samples of individuals from Uganda, Germany and the USA (non-endemic areas for *brugian* filariasis), and showed promising results with sensitivity and specificity of 100%. The objective of this study was to evaluate the novel diagnostic tool in the field by comparing its sensitivity to the conventional/standard diagnostic tool, namely microscopic examination of thick blood smears. The study was conducted in Belitung Timur district, a *B. malayi* endemic area, where the local LF elimination program started in 2005.

## Methodology

### Study approval

Verbal informed consent was obtained from all adult participants. For child participants, verbal assent was obtained from their legal guardians, teacher, and the head of Primary Health Center. This study received an ethical approval from Universitas Indonesia (Approval No. KET-631/UN2.F1/ETIK/PPM.00.02/2023).

### Study area

Belitung Timur district, an endemic area for LF caused by *B. malayi*, has been the focus of LF elimination through MDA with a combination drug of DEC and albendazole since 2005. Despite these efforts, the district failed to pass the Transmission Assessment Survey (TAS) in 2014 using IgG4 antibody detection, *Brugia* Rapid Test, in schoolchildren, prompting two additional rounds of DEC-albendazole treatments. Subsequent evaluations, including pre-TAS assessments using night thick blood smears, followed by TAS (1 and 2) assessments through detection of IgG4 antibodies in first- and second-grade schoolchildren (age 6–8), demonstrated that Belitung Timur successfully passed these evaluations. However, the district was unable to proceed to TAS 3 due to the lack of reliable diagnostic tools for IgG4 antibody detection at that time [9,10].

**Fig 1:**
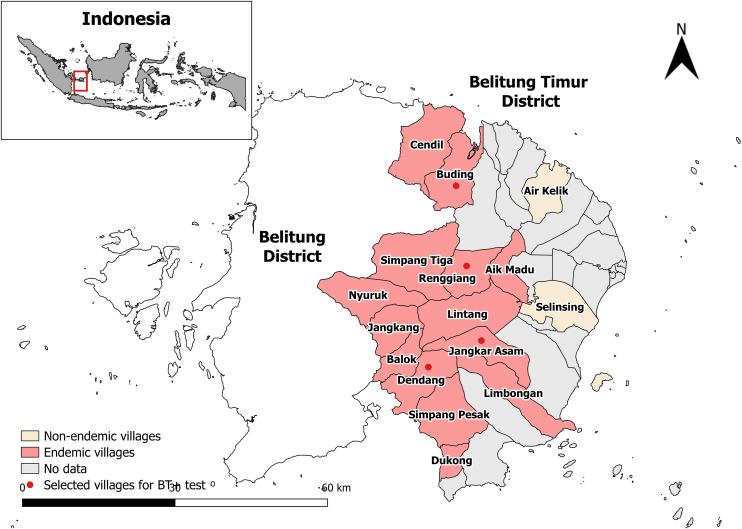
Map of the study area in Belitung Timur District, Indonesia. Belitung Island is divided into two districts: Belitung District (left) and Belitung Timur District (right). The study area, Belitung Timur District, includes sixteen labeled study villages. Among these, fourteen were classified as endemic (highlighted in pink) and two were non-endemic (in yellow). From the endemic villages, four high-endemicity villages were chosen for the *Brugia* Test Plus diagnostic assessment (red dots). Base map layer sourced from GADM for Indonesia (https://gadm.org/download_country.html, select “Indonesia”) and used under the GADM license (https://gadm.org/license.html). Map created using QGIS.

Belitung Timur district consists of 39 villages with a total population of 132,355. A preliminary survey (Survey 1) was conducted in May 2023 in 16 villages across six primary health centres (Fig 1). These villages were strategically selected due to their proximity to other *B. malayi* endemic areas in the neighbouring Belitung district, making them important locations for assessing the success of LF elimination efforts. Out of the 16 surveyed villages, 14 were identified as endemic by thick blood smear from night blood collection (Table 1).

**Table 1:** Crude prevalence of lymphatic filariasis in 16 villages of Belitung Timur district.

Primary Health Center	Village	No. Mf+	Total sample	Prevalence (%)
Damar	Air Kelik	0	115	0
Dendang	Balok	5	328	1.52
	Dendang	6	100	6.00
	Jangkang	6	615	0.98
	Nyuruk	7	472	1.48
Gantung	Jangkar Asam	5	139	3.6
	Limbongan	3	308	0.97
	Selinsing	0	217	0
Kelapa Kampit	Buding	3	135	2.22
	Cendil	4	171	2.34
Simpang Pesak	Dukong	2	141	1.42
	Simpang Pesak	2	126	1.59
Simpang Renggiang	Air Madu	7	185	3.78
	Lintang	6	492	1.22
	Renggiang	3	133	2.26
	Simpang Tiga	2	116	1.72
Total		61	3793	1.61

Further night blood collection in June 2023 (Survey 2) was conducted in four endemic villages -Buding, Renggiang, Jangkar Asam, and Dendang – where Mf prevalence exceeded 1% [11]. The study aimed to collect samples from the populations living in the LF endemic areas, targeting at least 300 individuals per village based on the minimum sample size required for pre-TAS [11]. Participants were distributed across household groups knows as Rukun Tetangga (RT), the smallest administrative unit within the village governance system. For example, in a village with 10 RTs (RT 1 to RT 10), 30 participants were randomly selected from each RT.

### Blood sample collection

This study employed two different sample collection methods: night blood collection targeted communities and day blood collection targeted schoolchildren.

#### Night blood collection.

For community participants, approximatively 250 µL of night blood was collected between 8:00 PM and 12:00 AM by a finger pick and stored in an EDTA tube (Microtainer Tube, BD, New Jersey, USA). From this sample, 60 µL was used to prepare a thick smear for microscopic examination, which increase the chance of detecting microfilariae, especially important in areas where MDA has been conducted [11]. The remaining blood was centrifuged to separate the plasma, which was then transferred into new tubes and stored at -20°C. These plasma samples were transported to Jakarta under cold conditions and stored at -20°C in the laboratories of the Department of Parasitology, Faculty of Medicine, Universitas Indonesia, for IgG4 antibody detection.

All microfilaremic patients identified in the preliminary survey were included in the night blood collection for this study. Those samples (n = 61) with additional Mf-positive individuals found in this study (n = 12) were recruited for a sensitivity assay, comparing the presence of Mf detected by microscopy of blood smears and IgG4 antibody detection using the BT+ test.

#### Day blood collection.

The dried blood spot (DBS) technique was used to collect samples from grade 1 and grade 2 elementary school children living in four selected villages (Buding, Renggiang, Jangkar Asam, and Dendang). A total of 244 children from seven elementary schools participated in the blood collection. Samples were taken during the daytime (8:00 AM–10:00 AM) at their schools. Blood was drawn by puncturing a fingertip with a sterile disposable lancet. The first drop of blood was wiped away with a gauze pad to remove excess tissue fluid. The fingertip was then massaged to stimulate blood flow, and the next drop was transferred onto one of the circles on a labelled filter disk (TropBio cat. no. FP). The blood was absorbed into the filter by capillary action, and this process was repeated to create six blood spots per child, each spot containing 10 µL of blood. Once air-dried, the filters were stored individually in plastic bags with desiccants at room temperature and then stored at -20°C for further use.

### Thick Blood Smear

Three lines of blood smear were prepared on a slide from each sample, with each line containing 20 µl of blood [11]. After air-drying, the blood was haemolysed, fixed with methanol, and stained with Giemsa. The stained slides were examined under a microscope to determine the Mf count in 60 µl of blood. Two independent technicians at Universitas Indonesia were assigned to examine the slides. The Mf count was reported based on the total number of Mf found in three blood lines.

### IgG4 antibody detection by *Brugia* Test Plus

#### Whole blood samples.

For the sensitivity testing of BT+ , we compared fresh whole blood, EDTA-treated blood, and plasma samples to evaluate two factors: first, whether the use of EDTA as an anticoagulant affects the sensitivity of BT+ ; and second, whether 2.5 µL of plasma provides comparable sensitivity to 5 µL of fresh whole blood. Thick blood smears with known microfilariae (Mf) counts, confirmed by microscopic examination, were used as the gold standard. A total of 10 Mf-positive samples were included in this evaluation.

#### Plasma samples.

The *Brugia* Test Plus (BT+) was used to detect the presence of anti-filarial IgG4 antibodies in plasma samples from the population. The test was conducted at the Laboratory of Parasitology, Universitas Indonesia, Jakarta, in accordance with the manufacturer’s instructions. A 2.5 µL volume of plasma was applied to the sample port, followed by the addition of three drops of buffer solution to the rectangular buffer port. To ensure accuracy and consistency, the results were read after 25 minutes by three independent readers, each within a three-minute window. A result was considered positive or negative when two of the three readers provided the same binary interpretation. In addition, we visually graded the intensity of the test line using a simple ordinal scale (absent, shadow, weak, medium, strong). Given concerns about reduced sensitivity in low-level infections during post-MDA surveillance, this grading was used to explore whether line strength could act as a proxy for infection intensity and to assess test performance at low Mf counts.

#### Dried blood samples.

A 96-well plate was used to elute the dried blood spot samples. 100 µL of chase buffer was added into each well of the plate, and then 1 circle of dried blood spot from each individual was added to each well. After incubating for one hour at room temperature, 10 µL of eluted dried blood spot (DBS) was added to the sample port. Three drops of buffer solution were then added to the rectangular buffer port. The results were read after 25 minutes of incubation by three independent readers, following the same procedure used for the plasma samples.

### Statistical analysis

Data, including name, gender, age, village name, Mf count, and BT+ result, were entered into an Excel file. Statistical analysis was performed using SPSS version 20. The comparison between the prevalence rates of Mf and IgG4 tested by BT+ was conducted using the McNemar test. The agreement among three readers who read the BT+ results was analysed using Cohen’s kappa agreements.

## Results

**Fig 2:**
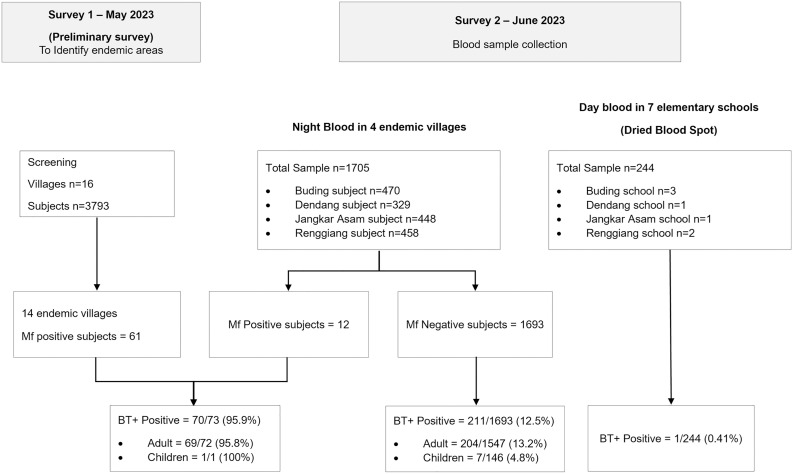
The flow of study participants in Belitung Timur.

Sixteen villages in Belitung Timur were screened using night blood smears to assess the Mf prevalence. Most of these villages are located near the Belitung district. The flow of study participants is shown in Fig 2 (Survey 1). The results from preliminary survey revealed that 14 out of the 16 villages are endemic for LF (Table 1). A total of 61 Mf-positive patients were identified in these 14 endemic villages, comprising 60 adults and one 14-year-old boy.

Two villages, Limbongan and Jangkang, had the lowest Mf rates, at 0.97% and 0.98%, respectively. The Mf prevalences in the remaining 12 villages ranged from 1.22% to 6%. For this study, four endemic villages were chosen: Dendang (6% Mf rate), Jangkar Asam (3.6%), Buding (2.22%), and Renggiang (2.26%). The selection of these villages was based on the presence of elementary schools, which facilitate the collecting of the day blood samples from schoolchildren.

### Characteristics of samples

A night blood collection was conducted among residents of four endemic villages (1,705 individuals, Table 2) including 61 individuals identified as Mf+ in a previous survey (Table 1). Among these individuals, 907 (51.4%) were male and 859 (48.6%) were female.

**Table 2:** Percentage of Mf+ in each endemic village.

Primary Health Center	Village	Children (≤ 17 years)	Adults (> 17 years)	No. Mf+ (Prevalence %)	Total
Dendang	Dendang	42 (12.77%)	287 (87.23%)	2 (0.61%)	329
Gantung	Jangkar Asam	42 (9.38%)	406 (90.63%)	3 (0.67%)	448
Kelapa Kampit	Buding	32 (6.81%)	438 (93.19%)	4 (0.85%)	470
Simpang Renggiang	Renggiang	30 (6.55%)	428 (93.45%)	3 (0.66%)	458
Total		146 (8.56%)	1559 (91.44%)	12 (0.70%)	1705

Microscopic examination of the population revealed that 12 out of the 1,705 individuals were Mf-positive, resulting in the Mf rate ranging from 0.61% to 0.85% (Table 2). All microfilaremic patients were adults and were distributed across the villages as follows: Dendang (2 patients), Jangkar Asam (3 patients), Buding (4 patients), and Renggiang (3 patients). No Mf-positive children were observed in those four endemic villages.

### Sensitivity of BT+ using whole blood, with or without EDTA

**Fig 3:**
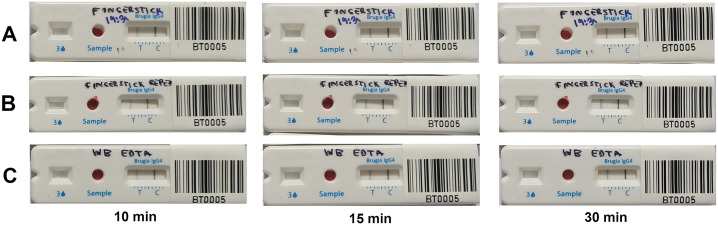
Comparison of Fingerstick blood with and without anticoagulant on the same subject. Row A and B show BT+ from blood samples without anticoagulant in duplicates while row C shows BT+ from EDTA-treated blood.

**Fig 4:**
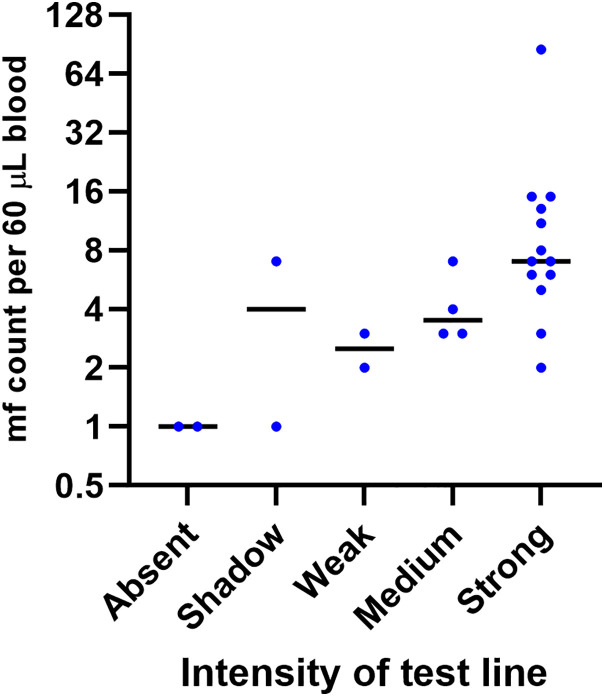
Appearance of the test line when run with fresh blood, without anticoagulants, compared to Mf density.

The BT+ test was evaluated in the field as per manufacturer’s instructions (S1 Fig) using fresh fingerstick blood, devoid of any anticoagulant, from 23 Mf-positive persons with Mf densities ranging from 1 Mf/60 µL to 85 Mf/60 µL. A total of 19 of 23 patients showed anti-filarial IgG4 antibodies. Another 10 Mf-positive individuals had further tests done using freshly collected fingerstick blood, but this time with EDTA as anticoagulant. Of these, 9/10 patients were positive for BT+. Furthermore, on a limited number of patients, it was shown that whole blood behaves similarly whether containing EDTA or no anticoagulant (Fig 3). Hence, when combining the data without (n = 23) and with EDTA (n = 10), the sensitivity of the BT+ assay was 28/33, or 84.9% (95% CI 68.1-94.9, n = 33). The assay detected antibodies in all the samples that had at least 2 Mf/60 µL and had difficulties detecting only 1 Mf/60 µL (Fig 4).

### Sensitivity of BT+ when using plasma (from EDTA blood)

A total of 73 sera samples from Mf-positive individuals identified by microscopic examination of nighttime thick blood smear (61 Mf-positive cases from previous survey and 12 Mf-positive cases from this study, as shown in Tables 1 and 2), consisting of 61 males and 12 females, were tested for the presence of anti-filarial IgG4 antibodies using the BT+ assay (Table 3).

**Table 3:** Percentage of anti-filarial IgG4 positivity in microfilaremic patients based on gender.

Mf count per 60 µL	Female			Male		
	Mf+	BT+ Positive	BT+ Negative	Mf+	BT+ Positive	BT+ Negative
1–10	7	7	0	30	28	2
11–20	2	2	0	15	14	1
21–30	2	2	0	7	7	0
31–40	0	0	0	4	4	0
50–100	1	1	0	4	4	0
>100	0	0	0	1	1	0
Total %	12	12 (100%)	0 (0%)	61	58 (95.1%)	3 (4.9%)

The Mf counts among the participants varied from 1 Mf/60 µL to 129 Mf/60 µL. Of these, four patients had a low Mf count of 1 Mf/60 µL; two of these low Mf patients tested positive for IgG4 antibodies by the BT+ assay, while the other two showed a negative result. Additionally, one patient with an Mf count of 12 Mf/60 µL also showed negative for IgG4 antibodies. All three IgG4-negative cases were male participants, whereas all female Mf-positive participants tested positive for IgG4 antibodies.

In total, 3 out of the 73 microfilaremic patients (4%) were negative for IgG4 detection by BT+ , with two of the three negatives only having 1 Mf/60 µL by microscopy. Overall, this study showed that the BT+ assay successfully detected anti-filarial IgG4 antibodies in 70 out of 73 microfilaremic patients, resulting in a sensitivity of 96%. These findings demonstrate the high sensitivity of the BT+ assay in identifying *B. malayi* infections.

**Fig 5:**
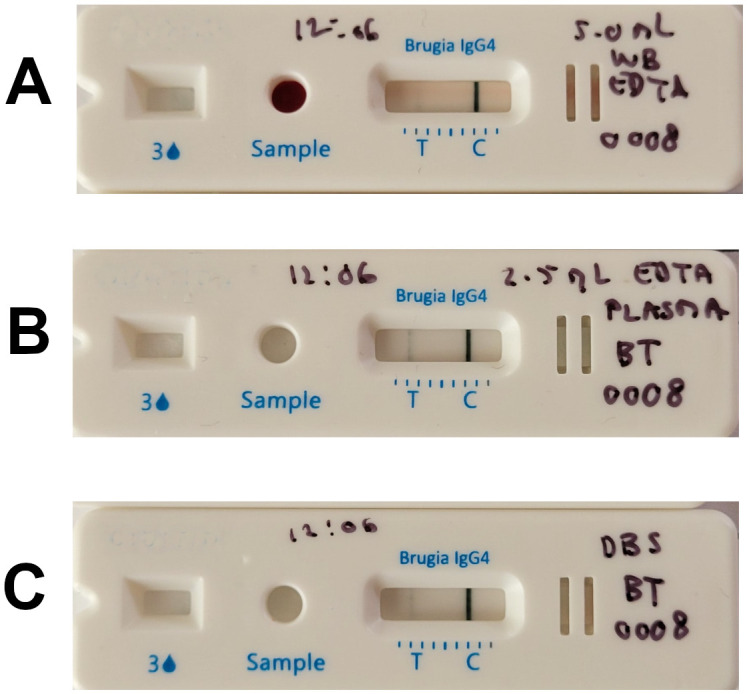
Comparison of 3 matrices from the same patient with EDTA whole blood (A), EDTA plasma (B), and DBS (C). Whole blood and DBS give very tenuous test lines, while plasma gives a more easily visible test line.

It should be noted that although the study was not powered to provide statistically significant data regarding matrix equivalence, it appears that when close to the limit of detection, the sensitivity is somewhat better with plasma than with DBS or whole blood (Fig 5).

### Detection of IgG4 antibody in all Mf negative samples

BT+ was also used to detect the presence of anti-filarial IgG4 antibodies in all Mf-negative participants from the study population. A total of 1,693 samples from 146 children aged 6–17 years old and 1,547 adults aged 18–90 years old, who were confirmed Mf-negative by microscopic examination of thick blood smears, were examined for the positivity of IgG4 antibodies. The results showed that 211 samples (7 from children aged 10–16 years old and 204 from adults) were positive for IgG4 antibodies using the BT+ assay. This indicated a prevalence of IgG4 positivity of 4.79% (7/146) in children and 13.19% (204/1,547) in adults (Table 4).

**Table 4:** Percentage of IgG4 positivity in Mf negative individuals.

Village	Children (<=17 Years)		Adults (>17 Years)		Total (%)
	Mf Negative	BT+ Positive (%)	Mf Negative	BT+ Positive (%)	
Dendang	42	1 (2.4%)	285	30 (10.5%)	327 (9.5%)
Jangkar Asam	42	1 (2.4%)	403	54 (13.4%)	445 (12.4%)
Buding	32	4 (12.5%)	434	61 (14.1%)	466 (13.9%)
Renggiang	30	1 (3.3%)	425	59 (13.9%)	455 (13.2%)
Total	146	7 (4.8%)	1547	204 (13.2%)	1693 (12.5%)

### Detection of IgG4 antibodies from dried blood spot samples of school children

A total of 244 grade 1 and grade 2 schoolchildren (age 6–8 years) from seven elementary schools participated in the day blood collection. The BT+ results revealed that only one child from Dendang’s elementary school tested positive for IgG4 antibodies (Table 5).

**Table 5:** *Brugia* Test Plus results from seven elementary schools.

Village	School	BT+ Positive	BT+ Negative	Total
Buding	SDN 1 Kelapa Kampit	–	29	29
	SDN 10 Kelapa Kampit	–	24	24
	SDN 7 Kelapa Kampit	–	36	36
Dendang	SDN 1 Dendang	1	53	54
Jangkar Asam	SDN 15 Gantung	–	59	59
Renggiang	SDN 5 Renggiang	–	24	24
	SDN 6 Renggiang	–	18	18
Total		1 (0.41%)	243 (99.59%)	244 (0.41%)

There were nine school children—three from Dendang Village, one from Jangkar Asam village, and five from Renggiang Village—who also participated in the night blood collection. The BT+ test using 2.5 µL of plasma yielded the same negative results as the BT+ test using DBS samples.

### Agreement in reading the *Brugia* Test Plus among three readers

In this study, three independent readers (Table 6) were assigned to interpret the results of the BT+ . Out of the 1,766 samples tested with BT+ , only 27 samples (1.53%) showed differences in reader interpretation either positively or negatively. Crosstabs statistical analysis was used to calculate the agreement among the readers. The results showed that the kappa agreement value between readers 1 and 2 was 0.964, between readers 1 and 3 was 0.945, and between readers 2 and 3 was 0.977. The overall agreement among all raters was 0.962 as measured by Fleiss’ kappa, indicating almost perfect agreement.

**Table 6:** Pairwise inter-reader agreement in BT+ interpretation.

		Reader 1		Reader 2		Reader 3	
		BT+ Pos	BT+ Neg	BT+ Pos	BT+ Neg	BT+ Pos	BT+ Neg
**Reader 1**	**BT+ Pos**			271 (15.3%)	6 (0.3%)	266 (15.1%)	11 (0.6%)
	**BT+ Neg**			11 (0.6%)	1478 (83.7%)	15 (15%)	1474 (83.5%)
**Reader 2**	**BT+ Pos**					276 (15.6%)	6 (0.3%)
	**BT+ Neg**					5 (0.3%)	1479 (83.7%)

## Discussion

In regions where *brugian* filariasis is endemic, an anti-filarial IgG4 antibody test is important to monitor the success of elimination programs, since it shows past and/or recent infection.

The utility of detecting anti-filarial IgG4 antibodies to BmR1 was first demonstrated by Noordin et al more than 20 years ago [8]. A rapid test based on that technology and commercialized by Reszon Diagnostics was investigated in multicentre studies and showed 99% specificity and 95% sensitivity for detecting IgG4 antibodies in *B. malayi*-infected samples [8,12]. Field evaluations comparing the *Brugia* Rapid Test with microscopic examination of thick blood smears revealed a sensitivity of 87% and a specificity of 100% [13].

The IgG4 antibody detection can be used in LF-endemic areas, whether caused by *Brugia malayi* or *Brugia timori*. Several studies have demonstrated the advantages of IgG4 antibody detection in assessing the endemicity of *brugian*-filariasis. For instance, research conducted in *B. malayi* endemic areas comparing micropore filtration (examined under a microscope) with IgG4 antibody detection showed that when Mf prevalence by microscopy was 6–7%, the seroprevalence was 46.9%. This demonstrates its ability to detect previous or amicrofilaremic infections [14]. Similarly, a study in *B. timori* endemic areas in Alor District revealed an 81% seroprevalence using IgG4 antibody detection, compared to 26% Mf prevalence by the nucleopore filtration method [15]. These discrepancies between IgG4 results and Mf prevalence are to be expected and demonstrate the sensitivity of IgG4 testing in detecting very low parasite loads or single-sex (only male or female) worm infections, as well as cryptic infections, past and/or recent infection. Furthermore, a serological test can be performed using blood collected during the day, which is a major programmatic advantage over collecting nighttime blood. These findings indicated the usefulness of the IgG4 test in evaluating the progress of filariasis elimination efforts [6,16].

Although the original test was easy to use in the field, there have been issues with sensitivity varying across different production batches. This inconsistency has made it difficult for program managers to rely on the test for monitoring elimination programs in *brugian* filariasis-endemic areas. The BT+ test, with a sensitivity of 96% compared to Mf positive subjects, is a novel assay that detects IgG4 specific for BmR1 and offers an alternative method for testing.

The BT+ assay was able to identify the existence of anti-filarial IgG4 antibody in microfilaremic patients with the lowest Mf count of 1–2 Mf/60 µL, but the sensitivity was only 33%. The fact that the test is sensitive even in samples containing only 1–2 Mf/60 µL seems to justify its use in areas of low endemicity. Taken together, these findings suggest that the BT+ assay will be essential for evaluating the effectiveness of the elimination program after mass drug administration (MDA) in endemic areas.

From an epidemiological standpoint, among 1,693 Mf-negative individuals, 211 individuals were anti-filarial IgG4 antibody positive (12.46%). Among the IgG4 positive individuals, adults were more likely positive compared to children. Nearly all the positive children were 10–16 years old; only one was 6–8 years old. Therefore, it appears that the risk of exposure to filarial infections increases with age. As a result, in endemic areas, adults have a higher percentage of positive BT+ than 10–16-year-old children, which in turn have a higher seroprevalence than 6–8-year-old children who are the target population of TAS [17]. This finding raises questions with regard to the optimal age group to be used as sentinel groups to evaluate effectiveness of the elimination program, and adults or older children may be a better sentinel group than young children.

Many endemic areas for lymphatic filariasis (LF) are difficult to access, and transporting blood samples from these locations poses significant challenges. The development of the dried blood spot (DBS) method offers a practical alternative to traditional blood collection methods, such as microtainers or vacutainers. In this study, filarial IgG4 antibodies were successfully detected in day blood samples collected on DBS from schoolchildren. A previous study conducted in Alor, an area endemic to *Brugia timori*, supported these findings by demonstrating a higher rate of filarial IgG4 antibodies detected on DBS in 17 individuals, of whom 8 individuals were Mf-positive and 9 were PCR-positive. These findings underscore the simplicity and scalability of the DBS method for sample collection in elimination programs, as well as the effectiveness of BT+ in detecting filarial IgG4 antibodies in DBS.

Evaluating the level of agreement among the readers is essential to determine the reliability and ease of interpreting the BT+ results. This study showed high agreement among readers of BT+ results, demonstrating that the BT+ is easy to interpret visually by naked eyes, and indicating a high degree of consistency. These results support the usability of BT+ in diverse settings, reinforcing its reliability for detecting low levels of infection.

The new format diagnostic tool BT+ requires significantly less sample volume than the previous format for detecting anti-filarial IgG4 antibodies in both Mf-positive and Mf-negative individuals. It needs only a 5 µL blood sample or a 2.5 µL plasma/serum sample [18]. In addition to the reduced sample volume, the BT+ assay is easy to perform and to interpret the results based on high contrast test lines. These advantages make BT+ a more practical and efficient tool for detecting filarial infections, particularly after mass drug administration in *brugian* filariasis-endemic areas.

A limitation of this study is that the stability of test under higher temperature for transporting and storage of test to/in the field need to be tested since the endemic areas are usually in hot climate (more than 30°C), which has exceeded the maximum temperature allowed by the manufacturer.

## Supporting information

S1 Fig Details of *Brugia* Test Plus test procedure. Instructions for use with whole blood and dried blood spot (DBS) samples.
(PDF)

S1 Data Survey data. This file contains data from survey 2 (communities and school children) used for figures and tables.
(XLSX)
