# Supplementary material for: Validation of the Brugia Test Plus to detect IgG4 antibodies in individuals from Belitung Timur, a Brugia malayi endemic area in Indonesia
Source: PLoS Negl Trop Dis. 2025 Aug 20;19(8):e0013449. doi: 10.1371/journal.pntd.0013449 (PMC12367139; doi:10.1371/journal.pntd.0013449)
Supplement: S1 Fig — Instructions for use with whole blood and dried blood spot (DBS) samples. (PDF) [file pntd.0013449.s001.pdf]

TEST PROCEDURE FOR BLOOD

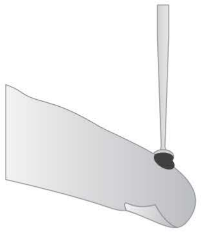

Place inverted cup in contact with blood.

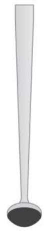

A droplet of blood will stay inside the cup.

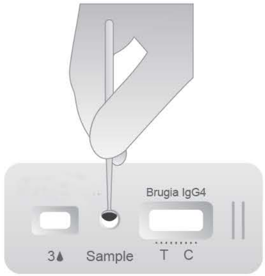

Round port – deposit blood with a light touch, without applying force (can damage pad).

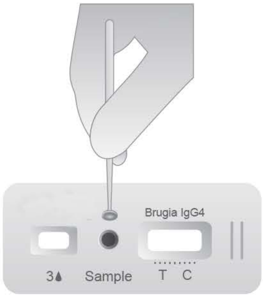

Lift cup gently. The sample area should be coated in blood. It is normal if a small amount of blood stays inside the cup.

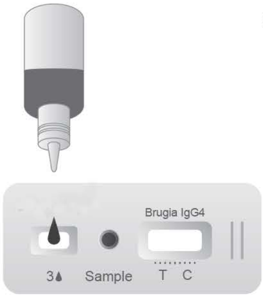

Rectangular port – add **3 drops** of buffer.

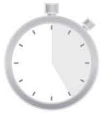

Wait **25 minutes**.

TEST INTERPRETATION

Positive

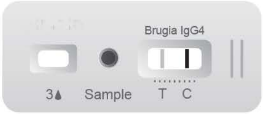

Test line (T) and control line (C) are both visible

Negative

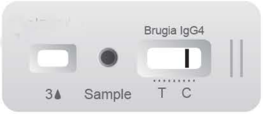

Only the control line (C) in the result window is visible

Invalid

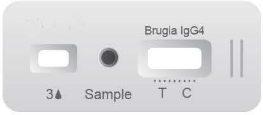

No control line (C) in the result window

Lines should be clearly visible. If you are not sure if you are seeing a line, don't count it.

Note 1: Record results using the form on the back of this sheet, and take a picture of the tests for your own records.

Note 2: Avoid shaking bottle – the buffer is soapy and makes bubbles.

Note 3: If using a micropipette, transfer 5 μL of blood or 2.5 μL of plasma/serum.

TEST PROCEDURE FOR DRIED BLOOD SPOTS

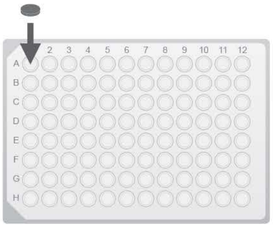

Place dried blood spot in well of untreated 96-well plate.

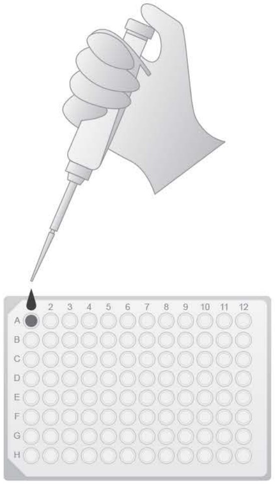

Add 100 μL of chase buffer to well. Incubate 1 h at ambient temperature.

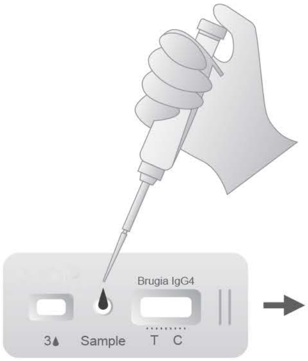

Transfer 10 μL to round sample port.

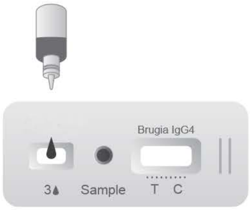

Add 3 drops of buffer to rectangular port.

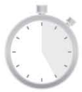

Read after 25 min. and interpret as above.
